# Choline Chloride/Lactic Acid Natural Deep Eutectic Solvent: A Multifunctional Tool for the Preparation of Chitosan Nanoparticles and Hybrid Nanocomposite Xanthan Gum Eutectogels

**DOI:** 10.3390/polym18202502

**Published:** 2026-10-13

**Authors:** Ioanna Pitterou, Kyriakos Kyriakopoulos, Maria-Aspasia Kosma, Evangelos Meletis, Andromachi Tzani, Sotirios P. Fortis, Anastasios G. Kriebardis, Anastasia Detsi

**Affiliations:** 1 Laboratory of Organic Chemistry, School of Chemical Engineering, National Technical University of Athens, Zografou Campus, 15772 Athens, Greece; 2 Laboratory of Reliability and Quality Control in Laboratory Hematology, Department of Biomedical Sciences, School of Health and Caring Sciences, University of West Attica, 12243 Athens, Greecesfortis@uniwa.gr (S.P.F.); akrieb@uniwa.gr (A.G.K.)

**Keywords:** eutectogels, NADES, chrysin, chitosan nanoparticles, green nanotechnology, hemocompatibility

## Abstract

In this work, an innovative hybrid eutectogel-chitosan nanoparticles system was developed, suitable for biomedical applications. Chitosan (CS) was used as a matrix for the encapsulation of the naturally occurring flavonoid chrysin (Chr), known for its significant biological activities but with limited applications due to its very low aqueous solubility resulting in poor oral bioavailability. A natural deep eutectic solvent (NADES), namely choline chloride/lactic acid at a 1:2 molar ratio, was used as a multifunctional component in the preparation of chitosan nanoparticles (CS-NPs) for chrysin encapsulation, facilitating nanoparticle formation under the investigated conditions. The hydrodynamic diameter of the CS-Chr NPs was 138.8 nm, as determined using nanoparticle tracking analysis, with a high positive ζ-potential of +46.53 mV. The encapsulation efficiency of the CS-Chr NPs was 45%. CS-Chr NPs and blank-CS NPs were evaluated for their hemolytic properties and demonstrated hemolytic activity, within the limits of allowed nanotoxicity (hemolysis < 5%). Additionally, the NADES acted as a matrix, with xanthan gum functioning as a gelator, for the preparation of eutectogels. The resulting eutectogel was developed to combine the advantages of the NADES-based environment and chitosan–chrysin nanoparticles (CS–Chr NPs) with the structural integrity and prolonged retention provided by a gel matrix, thereby offering a stable and versatile platform for the localized delivery of chrysin. The prepared xanthan gum eutectogels showed significant swelling ratio capacity (120%) even after the swollen sample was kept in the aqueous HCl solution (pH 1.2) for 3 h, indicating a good stability of the network. The release of chrysin from CS-Chr NPs reached 94% after 8 h, and exhibited predominantly Fickian diffusion (*n* = 0.4099). Incorporation of the CS-Chr NPs into the xanthan-gum-based eutectogel resulted in a more sustained chrysin release (76% after 8 h). The eutectogel release was predominantly diffusion-driven (Higuchi, R^2^ = 0.9796), with anomalous transport (*n* = 0.7247) indicating an additional contribution of polymer relaxation and/or swelling. The proposed NADES-based platform provides a sustainable strategy for the development of multifunctional nanocomposite eutectogels, combining biocompatible components with the unique functional properties of natural deep eutectic solvents for advanced biomedical delivery applications.

## 1. Introduction

**Figure 1:**
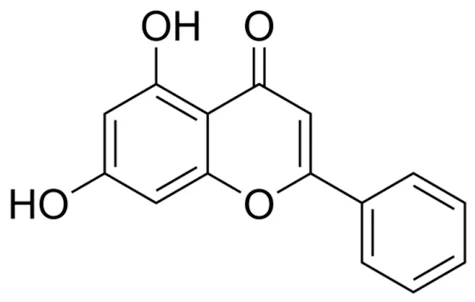
Chemical structure of chrysin.

Chrysin (Chr) (5,7-dihydroxyflavone) is a naturally occurring flavone found in various plant species, honeycomb, and passion fruit (Figure 1). It exhibits a broad spectrum of biological activities, including antioxidant, anticancer, antidiabetic, anti-inflammatory, and neuroprotective effects [1]. However, despite its promising therapeutic potential, the practical biomedical application of chrysin remains limited due to its poor aqueous solubility, low bioavailability, and rapid metabolism [1,2]. Therefore, the development of efficient delivery systems capable of improving its solubility, stability, and bioavailability represents an important research challenge [3].

Chitosan, a natural polysaccharide obtained from the deacetylation of chitin, has attracted considerable attention as an encapsulation matrix for bioactive compounds due to its exceptional biocompatibility, biodegradability, non-toxicity, and intrinsic antimicrobial properties [4]. Under acidic conditions, the protonated amino groups of chitosan provide a cationic character, enabling the formation of diverse nanostructures, including nanoparticles, microparticles, films, membranes, and fibers. This versatility allows effective interactions with negatively charged molecules and surfaces, supporting its broad application in the biomedical, pharmaceutical, agricultural, and environmental fields [5].

Despite its advantageous properties, chitosan exhibits limited aqueous solubility and requires acidic conditions for dissolution, and crosslinking agents such as sodium tripolyphosphate (TPP) for nanoparticle preparation. Therefore, the development of sustainable strategies for the fabrication of chitosan-based nanocarriers remains an important research challenge [6].

Hydrogels are three-dimensional hydrophilic polymeric networks capable of absorbing and retaining large amounts of water while maintaining their structural integrity. Due to their unique physicochemical properties, hydrogels have been extensively investigated as delivery platforms for therapeutic molecules [7]. The incorporation of nanoparticles into hydrogel matrices leads to the formation of hybrid nanocomposite systems that combine the advantageous characteristics of both components [8]. Such integration can overcome several limitations associated with nanoparticles, including potential toxicity, poor stability, and uncontrolled release of encapsulated compounds, while simultaneously improving the mechanical strength, structural stability, and functionality of hydrogels. Furthermore, these hybrid systems offer enhanced versatility regarding administration routes and biomedical applications [9,10].

Xanthan gum is a naturally derived microbial polysaccharide produced through the fermentation of *Xanthomonas campestris* and has gained considerable attention due to its biodegradability, biocompatibility, non-toxicity, and excellent stability over a wide range of pH and temperature conditions [11]. In addition, its cost-effectiveness and high efficiency at relatively low concentrations make it an attractive biomaterial for pharmaceutical, food, and cosmetic applications [12,13].

Natural deep eutectic solvents (NADES) have emerged as sustainable alternatives to conventional solvents due to their low toxicity, biodegradability, and ability to enhance the solubility and extraction efficiency of bioactive compounds [14,15]. Their multifunctional nature allows their utilization not only as green solvents but also as active components in the design of advanced biomaterials and nanostructured delivery systems [16].

Eutectogels represent an emerging class of gel-based materials consisting of a three-dimensional polymeric network capable of immobilizing a eutectic solvent, resulting in a non-flowing and self-supporting structure. By combining the unique properties of eutectic solvents, including low volatility, tunable physicochemical characteristics, biocompatibility, and high ionic conductivity, with the flexibility and mechanical adaptability of polymeric networks, eutectogels provide multifunctional platforms with promising applications in drug delivery, energy storage, and environmental technologies [17,18,19].

Due to its favorable characteristics, xanthan gum was selected as the polymeric network-forming/gelating agent for the development of the NADES-based eutectogel system, enabling the formation of a stable three-dimensional polymeric network capable of immobilizing the eutectic phase [13].

In the present study, the multifunctionality of NADES was exploited to simplify and enhance the sustainability of the developed hybrid delivery platform for chrysin (Chr) encapsulation. The choline chloride/lactic acid (ChCl/LA) NADES (molar ratio 1:2) was employed as a multifunctional component in the developed formulation: (i) lactic acid initially acted as the acidic medium facilitating chitosan protonation and dissolution; (ii) the incorporation of ChCl/LA NADES promoted non-covalent intermolecular interactions within the chitosan nanoparticle system, contributing to nanoparticle stabilization and facilitating the formation of CS-NPs without the need for conventional external ionic crosslinkers such as TPP; and (iii) NADES served as the eutectic liquid phase within the eutectogel structure, physically immobilized within the three-dimensional polymeric network formed by xanthan gum.

In this context, the present work introduces a sustainable hybrid nanocomposite eutectogel platform for the encapsulation and delivery of chrysin. This innovative system exploits the multifunctional role of NADES as both a green processing medium for the preparation of chitosan nanoparticles and an integral structural component of the eutectogel network. The proposed strategy combines nanoscale encapsulation, enhanced chrysin solubilization, improved nanoparticle stability, and a biocompatible gel-based matrix, enabling the development of an environmentally sustainable delivery platform with enhanced potential for pharmaceutical applications.

Hemolysis is defined as the disruption of the erythrocyte membrane and the subsequent release of hemoglobin and other intracellular components into the extracellular environment [20]. Excessive intravascular hemolysis may contribute to anemia, jaundice, oxidative and vascular injury, and renal dysfunction; therefore, evaluation of hemolytic potential represents an important aspect of the preliminary safety assessment of drug delivery systems intended for biomedical and pharmaceutical applications [21,22,23,24]. Nanoparticles may interact with erythrocyte membranes through electrostatic, hydrophobic, or mechanical mechanisms, and the extent of membrane damage may depend on particle size, surface charge, composition, aggregation state, concentration, and exposure time [22,25]. Even for formulations not primarily intended for intravenous administration, the hemolysis assay provides a sensitive initial indication of membrane compatibility in the event of systemic exposure to released nanoparticles.

## 2. Materials and Methods

### 2.1. Materials

Ultra-low-molecular-weight chitosan (viscosity < 5 cP, average molecular weight 20,000 Da) and xanthan gum from *Xanthomonas campestris* (G1253, Mw ≈ 2000 kDa) were purchased from Sigma-Aldrich (Merck KGaA, Darmstadt, Germany). Choline chloride and chrysin were obtained from Alfa Aesar (Haverhill, MA, USA), while D,L-lactic acid (80% aqueous solution) was purchased from LabKem (Barcelona, Spain). Hydrochloric acid (37% *w/w*) was purchased from Fisher Chemical (Loughborough, UK). Ultrapure water (18.2 MΩ·cm at 25 °C) was used throughout all experiments.

### 2.2. NADES Preparation

The NADES was prepared using the heating and stirring method according to the procedure reported in our previous work [14]. Briefly, choline chloride (ChCl) (10.2 g, 73.1 mmol) and D,L-lactic acid (LA) (16.4 g, 182.1 mmol) were mixed at a molar ratio of ChCl/LA 1:2. The components were transferred into a round-bottomed flask and heated at 60 °C under continuous magnetic stirring until a clear homogeneous liquid was formed (approximately 2 h). The obtained NADES was used without further purification.

### 2.3. Preparation of Chitosan Nanoparticles (CS NPs)

Initially, a 2% (*v*/*v*) aqueous D,L-lactic acid (LA) solution was prepared in a round-bottom flask. Subsequently, 20 mg of chitosan (CS) was added under continuous stirring until complete dissolution was achieved, yielding a final CS concentration of 0.2% (*w*/*v*) in a total volume of 10 mL. A 10% (*w*/*v*) NaOH solution was then gradually added to the chitosan solution to adjust the pH to 4.8. The obtained dispersion was vacuum-filtered to remove any undissolved impurities and minimize aggregate formation.

For the preparation of chrysin-loaded chitosan nanoparticles (CS-Chr NPs), chrysin was initially dissolved in ethanol (EtOH), and the solvent was subsequently evaporated to obtain a chrysin residue prior to incorporation into the chitosan dispersion. The amount of chrysin was defined relative to the mass of chitosan used as the nanoparticle carrier (% *w*/*w*). Specifically, 2.72 mg of chrysin was used per 20 mg of chitosan, corresponding to a chrysin content of 13.6% (*w*/*w*, relative to chitosan mass). Subsequently, 30 μL of NADES (ChCl/LA, molar ratio 1:2), corresponding to a final concentration of 0.3% (*v*/*v*), was added. The NADES acted as a natural cross-linking/modifying agent, promoting nanoparticle formation and contributing to the stabilization of the chitosan-based system. The resulting CS-Chr NPs dispersion was magnetically stirred overnight to facilitate interactions among the components and obtain a homogeneous nanoparticle suspension.

Blank CS nanoparticles (Blank CS NPs) were prepared following the same procedure, without the addition of chrysin, in order to evaluate the contribution of the active compound to the physicochemical properties of the nanoparticle system.

### 2.4. Chitosan-Nanoparticles (CS NPs) Characterization

#### 2.4.1. Nanoparticle Tracking Analysis (NTA)

All experiments were performed using a NanoSight NS300 instrument (Malvern Instruments Ltd., Worcestershire, UK) equipped with a 532 nm green laser and a high-sensitivity scientific complementary metal-oxide-semiconductor (sCMOS) camera, both from Malvern Instruments Ltd. For each sample (DS and DP), three independent replicates were analyzed, with three 60 s videos recorded per replicate. A minimum of 1000 valid tracks was required to ensure the reliability of each experimental sample. The NTA acquisition was continuously monitored using a syringe loading system. The detection threshold was set at four for both labeled and unlabeled particles, and the background noise and camera level settings remained constant across all measurements. Data analysis was conducted using NanoSight NTA 3.1 software (Malvern Instruments Ltd., Worcestershire, UK).

#### 2.4.2. Polydispersity Index (PdI) and ζ-Potential

The polydispersity index (PdI) and ζ-potential of the synthesized nanoparticles were determined using a Zetasizer Nano ZS (Malvern Panalytical, Malvern, UK). For each measurement, 10 μL of the nanoparticle dispersion was diluted in 20 mL of ultrapure water and vortex-mixed for 2 min to ensure homogeneous dispersion. The diluted sample was transferred to a folded capillary cell (DTS1070, Malvern Panalytical, Malvern, UK), and the PdI was determined by dynamic light scattering (DLS), while the ζ-potential was measured by electrophoretic light scattering (ELS) at 25 °C. All measurements were performed in triplicate, and the results are reported as mean values.

#### 2.4.3. Transmission Electron Microscopy Study (TEM)

The samples under investigation were suspended in deionized water and subjected to ultrasonic treatment to disperse aggregated particles. Subsequently, a drop of the resulting suspension was placed on a copper grid coated with a 300 mesh carbon film, where it was allowed to air-dry overnight.

#### 2.4.4. Fourier-Transform Infrared Spectroscopy (FT-IR)

FTIR spectroscopy was performed on a JASCO FT/IR-4200 spectrometer (JASCO Corporation, Easton, MD, USA) in attenuated total reflectance (ATR) mode, in the range from 4000 to 400 cm^−1^.

#### 2.4.5. Thermogravimetric Analysis (TGA)

Thermogravimetric analysis (TGA) was performed on the CS-NADES NPs, chrysin, nanocomposite eutectogel, and blank eutectogel in a using a TGA/DSC 1 STARe System Thermobalance (Mettler Toledo, Columbus, OH, USA), with the samples being heated from 25 °C to 600 °C, at a heating rate of 10 °C/min under nitrogen gas flow (10 mL/min).

#### 2.4.6. Encapsulation Efficiency

To determine the encapsulation efficiency (EE%), the 10 mL CS-Chr NPs dispersion prepared as described in Section 2.3 was first freeze-dried. The obtained lyophilized nanoparticles were then dispersed in 10 mL of dimethyl sulfoxide (DMSO) and subjected to continuous magnetic stirring at room temperature for 48 h to ensure complete dissolution and release of the encapsulated chrysin. Following incubation, the absorbance of the resulting solution was measured using a UV–Vis/NIR spectrophotometer (Jasco V-770) at 270 nm. A corresponding blank sample containing DMSO and the CS/NADES matrix without chrysin was prepared and subjected to the same treatment under identical conditions. The absorbance of the blank was used for background correction to account for the contribution of DMSO and the formulation components. Chrysin concentration was determined using a calibration curve over the range of 0.001–0.005 mg/mL (A = 167.83C + 0.08596, R^2^ = 0.9952). The encapsulation efficiency (EE%) was calculated according to Equation (1):

(1) $$Encapsulation\;Efficiency\;\left(EE\%\right)=\frac{Experimental\;Chrysin\;content\;in\;NPs\;}{Theoretical\;Chrysin\;content\;in\;NPs}\times 100$$

#### 2.4.7. In Vitro Release Study of Chrysin from CS-Chr NPs

For the release study, 10 mL of the CS-Chr NPs dispersion, prepared as described in Section 2.3, was transferred into regenerated cellulose dialysis tubing (MWCO 12,000–14,000 Da; diameter 29 mm) and immersed in 60 mL of potassium phosphate buffer (pH 7.4) containing 1% (*v*/*v*) DMSO. The system was maintained at 37 °C under gentle agitation (100 rpm). At predetermined intervals (every 1 h), aliquots were withdrawn and replaced with an equal volume of fresh release medium. Chrysin concentration was determined by UV–Vis spectrophotometry at 270 nm using a calibration curve over 0.001–0.005 mg/mL (A = 167.83C + 0.08596, R^2^ = 0.9952). The method showed satisfactory recovery, LOD, LOQ, and selectivity, with no significant interference from the blank formulation or release medium. The release data were fitted to zero-order, first-order, Higuchi, and Korsmeyer–Peppas models, with the best-fitting model selected based on the highest R^2^. Experiments were performed in triplicate (n = 3), and results are expressed as mean ± SD.

#### 2.4.8. Hemolytic Assay

Peripheral venous blood samples were collected from 10 healthy adult volunteers in citrate blood tubes following written informed consent. A complete blood count was performed for each volunteer prior to inclusion, using the CONVERGYS^®^ X3 hematology analyzer. All measured hematological parameters were within the corresponding laboratory reference ranges, supporting the absence of overt hematological abnormalities. “The study was conducted in accordance with the Declaration of Helsinki and approved by the Research Ethics and Deontology Committee of the University of West Attica (protocol code 30581; date of approval: 21 March 2025).” Blood samples were processed within a few minutes of collection. Following centrifugation at 1000× *g* for 10 min, after which the plasma and buffy coat were removed. The isolated erythrocytes were subsequently resuspended in phosphate-buffered saline (PBS) to a final hematocrit of 5%. RBC suspensions were then incubated with either chrysin-loaded chitosan nanoparticles (CS-Chr NPs) or blank chitosan nanoparticles (CS-NPs). CS-Chr NPs were tested at a final chrysin concentration of 6 μg/mL. This concentration was prepared by a 1:20 dilution of the original 0.12 mg/mL CS-Chr NPs stock. RBCs suspended in PBS and deionized water served as negative and positive controls, respectively. Samples were incubated for 2 h at 37 °C under gentle agitation, followed by centrifugation at 10,000× *g* for 3 min. The absorbance of the supernatant was measured at 540 nm using a NOVASPEC spectrophotometer (Biochrom, UK) to determine the amount of released hemoglobin. The percentage of hemolysis [26] was calculated according to the following Equation (2):

(2) $$\mathrm{Hemolysis}\;(\%)\;=\;\frac{Abs\;sample\;-Abs\;negative\;control\;}{Abs\;positive\;control-Abs\;negative\;control}\times 100$$

### 2.5. Nanocomposite Eutectogel Synthesis

Xanthan gum (400 mg; 20% *w*/*v*) was used as the gelation agent and was added to 2 mL of NADES (ChCl/LA, 1:2 molar ratio). The vial containing the mixture was placed on a magnetic stirrer and heated at 80 °C for 30 min to facilitate the formation of the eutectogel network. After cooling the system to 60 °C, the CS-Chr NPs dispersion prepared as described in Section 2.3 was incorporated into the mixture at 10% (*v*/*v*) (222 μL) under continuous magnetic stirring to ensure homogeneous distribution throughout the matrix. Finally, the resulting nanocomposite eutectogels were freeze-dried and stored in a desiccator until further characterization.

Blank eutectogels (without nanoparticles) were synthesized using the same procedure, excluding the incorporation of any nanoparticle dispersion, in order to evaluate the effect of the CS-NPs and CS-Chr NPs on the structural and physicochemical properties of the eutectogel system.

**Figure 2:**
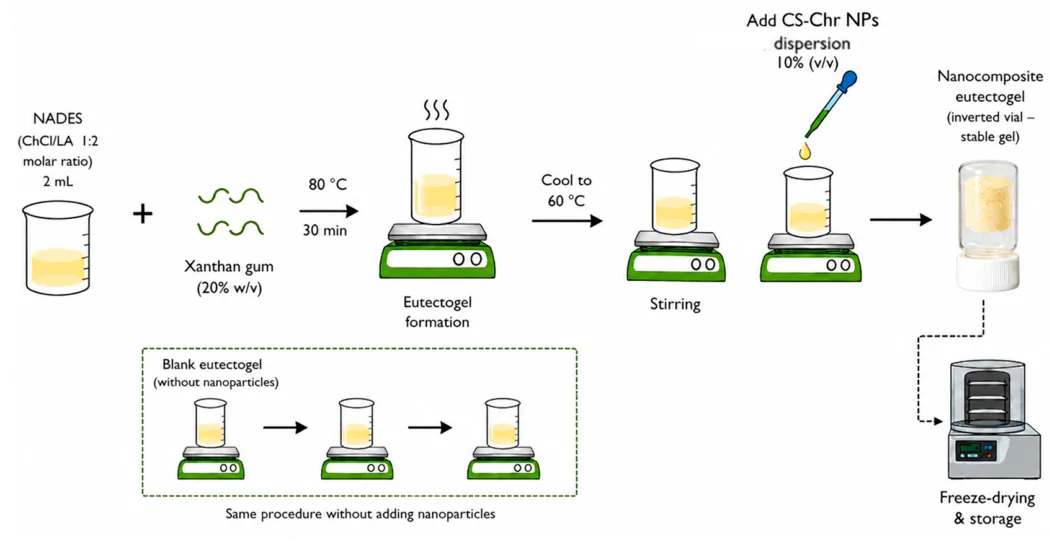
Schematic illustration of nanocomposite eutectogel preparation.

**Figure 3:**
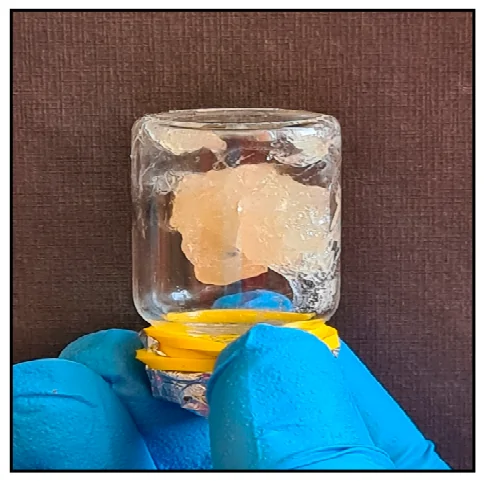
Macroscopic appearance of the hybrid nanocomposite eutectogel.

A schematic illustration of the preparation procedure for the nanocomposite eutectogel is presented in Figure 2. The macroscopic appearance of the hybrid nanocomposite eutectogel is shown in Figure 3.

### 2.6. Nanocomposite Eutectogel Characterization

#### 2.6.1. Swelling Ratio (SR%)

All eutectogels prepared for the swelling capacity (SR) experiment were first subjected to lyophilization. Subsequently, each lyophilized eutectogel was weighed (g) and transferred into a vial containing 10 mL of 0.1 M hydrochloric acid (HCl) solution (pH 1.2). The vials were then placed in an incubator set at 37 °C and 90 rpm. Weighing of the eutectogels was performed at regular intervals of 5, 10, 15, 20, 25, 30, 60, 120 and 180 min. At each time point, the eutectogel was removed from the vial, and excess surface HCl solution was carefully blotted with filter paper to ensure that the measurements reflected only the amount of HCl solution adsorbed by the eutectogel.

The swelling capacity, SR (%), was determined using Equation (3) below:

(3) $$SR\left(\%\right)=\frac{Ws-Wd}{Wd}\times 100$$

where SR (%) is the swelling ratio, W_s_ (g) is the mass of the swollen eutectogel at time t and W_d_ (g) is the mass of the dry eutectogel measured at t = 0.

#### 2.6.2. In Vitro Release Study of Chrysin from Nanocomposite Eutectogel

For the release study, 1.72 g of the hybrid nanocomposite eutectogel, prepared as described in Section 2.5, was immersed in 25 mL of potassium phosphate buffer (pH 7.4) containing 1% (*v*/*v*) DMSO and maintained at 37 °C under gentle agitation (100 rpm). At predetermined intervals (every 1 h), aliquots were withdrawn and replaced with an equal volume of fresh medium. Chrysin concentration was determined by UV–Vis spectrophotometry at 270 nm using a calibration curve over 0.001–0.005 mg/mL (A = 167.83C + 0.08596, R^2^ = 0.9952). The method showed satisfactory recovery, LOD, LOQ, and selectivity, with no significant interference from the blank formulation or release medium. The release data were fitted to zero-order, first-order, Higuchi, and Korsmeyer–Peppas models. The best-fitting model was selected based on the highest R^2^. Experiments were performed in triplicate (n = 3), and results are expressed as mean ± SD.

### 2.7. Impact of Independent Variables on Swelling Ratio of the Nanocomposite Eutectogel Formulation

The Box–Behnken experimental design was to investigate the effects of the most important factors (independent variables) on the swelling ratio of the nanocomposite eutectogel systems. For the experimental design the Design Expert^®^ 12.0 software (Stat-Ease Inc., Minneapolis, MN, USA—Trial Version) was used.

The parameters of the experimental design are given in Table 1. The concentrations of (A) xanthan gum, (B) chrysin and (C) CS NPs, were employed as the independent variables. The selected response was the (R1) swelling ratio (%) (at 3 h).

## 3. Results

The present study explored the potential of choline chloride/lactic acid (ChCl/LA) NADES (molar ratio 1:2) as a multifunctional platform for the development of chrysin-loaded chitosan- nanoparticles (CS-Chr NPs) designed for the encapsulation and delivery of chrysin, and their subsequent incorporation into eutectogels. Specifically, ChCl/LA NADES was employed as a multifunctional component within the developed formulation: (i) lactic acid, as an acidic component, facilitated chitosan protonation and dissolution, eliminating the need for additional acidic solvents; (ii) the incorporation of NADES into the chitosan dispersion promoted the organization and stabilization of the nanoparticle structure through molecular interactions between the eutectic components and the polymeric matrix, enabling the formation of CS-Chr NPs without the addition of conventional crosslinking agents such as sodium tripolyphosphate (TPP); and (iii) NADES served as the eutectic liquid phase during eutectogel fabrication, being immobilized within the xanthan-gum-based polymeric network, where xanthan gum acted as the network-forming/gelating agent. The selection of the NADES components was based on their biocompatibility and suitability for pharmaceutical and food-related applications, while chitosan and xanthan gum were selected due to their biodegradability, biocompatibility, and excellent gel-forming properties. Finally, the CS-Chr NPs were incorporated into the eutectogel matrix to develop an integrated delivery system with enhanced physicochemical characteristics and biomedical potential.

### 3.1. Characterization of CS-NADES NPs

#### 3.1.1. Hydrodynamic Diameter, Concentration, Polydispersity Index (PDI) and ζ-Potential

**Figure 4:**
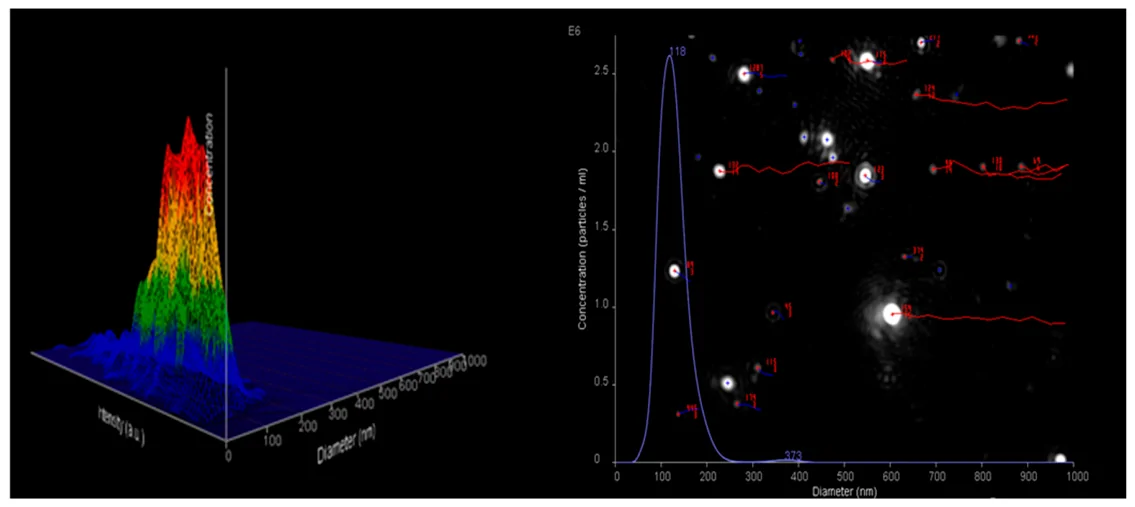
An indicative example of the size distribution profile of CS-Chr NPs generated by NTA.

Nanoparticle Tracking Analysis (NTA) is a highly sensitive and reliable technique that enables the determination of nanoparticle concentration and hydrodynamic diameter by tracking the Brownian motion of individual nanoparticles. Unlike ensemble-based techniques, NTA provides a more representative assessment of particle size distribution by analyzing individual particles. In addition, the polydispersity index (PDI) was determined by Dynamic Light Scattering (DLS) to assess the width and homogeneity of the nanoparticle size distribution. Lower PDI values indicate a narrower and more homogeneous particle size distribution. The ζ-potential of the nanoparticles was determined by Electrophoretic Light Scattering (ELS), a well-established technique that measures the electrophoretic mobility of suspended particles under an applied electric field and converts it into ζ-potential using the Henry equation. The ζ-potential provides valuable information regarding the surface charge and, consequently, the electrostatic stability of nanoparticle dispersions. The obtained results are presented in Table 2 and Figure 4.

Blank CS NPs exhibited an average hydrodynamic diameter of 134.6 ± 7.3 nm and a particle concentration of 1.38 × 10^7^ ± 2.19 × 10^6^ particles/mL, confirming the successful formation of nanosized chitosan-based structures. Following chrysin encapsulation, the hydrodynamic diameter increased only slightly to 138.8 ± 5.3 nm, while the particle concentration increased markedly to 5.54 × 10^7^ ± 2.79 × 10^6^ particles/mL. This limited increase in particle size suggests that chrysin incorporation did not substantially alter the nanoscale organization of the chitosan nanoparticles. The increase in particle concentration may be associated with changes in the nanoparticle population following chrysin incorporation and/or improved dispersion of the resulting nanosystems. Similar changes in the physicochemical characteristics of chitosan nanoparticles following chrysin incorporation have been reported by Siddhardha et al., who observed an increase in hydrodynamic diameter from approximately 299 to 355 nm and in PDI from 0.434 to 0.487 after chrysin loading [27].

The blank CS NPs exhibited a PDI value of 0.520 ± 0.03, while the CS-Chr NPs showed a slightly higher PDI of 0.565 ± 0.06, indicating relatively broad particle size distributions. The slight increase in PDI following chrysin incorporation is consistent with a modest broadening of the nanoparticle population upon drug loading. Importantly, the obtained particle size remains within the nanoscale range reported for chrysin-loaded chitosan nanoparticles prepared using conventional ionic gelation with sodium tripolyphosphate (TPP). Siddhardha et al. reported the successful preparation of chrysin-loaded chitosan nanoparticles using TPP as a crosslinking agent, demonstrating the suitability of chitosan-based nanosystems for chrysin delivery [27]. More recently, TPP-based chrysin-loaded chitosan nanoparticles with a mean particle size of approximately 199 nm were reported, further supporting the feasibility of chitosan to encapsulate chrysin [28]. In comparison, the hydrodynamic diameter of 138.8 ± 5.3 nm obtained in the present study demonstrates that the NADES-assisted approach can produce chrysin-loaded chitosan nanoparticles within a comparable nanoscale size range to conventional TPP-based systems [27,28].

The long-term stability of the CS-Chr NPs was further evaluated after 8 months of storage at 4 °C. A moderate increase in hydrodynamic diameter was observed, reaching 194.7 ± 28.5 nm, accompanied by an increase in PDI to 0.630 ± 0.04. The simultaneous increase in particle size and PDI suggests a certain broadening of the particle size distribution during storage, potentially associated with limited nanoparticle swelling, polymer-chain rearrangement, and/or mild aggregation. Nevertheless, the particle concentration remained comparable to that of the freshly prepared formulation (5.71 × 10^7^ ± 6.73 × 10^6^ particles/mL), indicating that no substantial loss of nanoparticles occurred during storage. Thus, although some changes in particle size distribution were observed, the nanoscale character of the system was retained over the 8-month storage period [29].

The ζ-potential of the nanoparticles was determined by Electrophoretic Light Scattering (ELS), and the corresponding values are presented in Table 2. Blank CS NPs exhibited a positive ζ-potential of +40.61 ± 1.25 mV, which increased slightly to +46.53 ± 2.61 mV following chrysin encapsulation. The maintenance of a strongly positive surface charge indicates that chrysin incorporation did not compromise the characteristic cationic nature of the chitosan nanoparticles. The positive ζ-potential can be attributed primarily to the protonated amino groups of chitosan exposed at the nanoparticle surface and may promote electrostatic repulsion between particles, thereby limiting aggregation [29]. Moreover, after 8 months of storage, the ζ-potential remained highly positive at +42.83 ± 2.13 mV, indicating preservation of the surface charge characteristics of the nanoparticles during storage.

The relatively high positive ζ-potential observed in the present study is also notable when compared with previously reported TPP-crosslinked chitosan–chrysin nanoparticles. For example, chrysin-loaded chitosan nanoparticles prepared by ionic gelation have been reported to exhibit a positive ζ-potential of approximately +22.9 Mv [28]. The higher ζ-potential observed for the present CS-Chr NPs (+46.53 ± 2.61 mV), prepared using NADES, may provide a stronger electrostatic contribution to colloidal stabilization. Although direct comparison should be interpreted cautiously because of differences in formulation composition, preparation conditions, and measurement protocols, the results demonstrate that the NADES-assisted strategy is capable of producing chitosan–chrysin nanoparticles with physicochemical characteristics comparable to, and in some respects more favorable than, those reported for conventional TPP-based systems [27,28].

Overall, the combination of nanoscale hydrodynamic diameter, positive surface charge, and reproducible particle concentration confirms the successful formation of the CS-Chr NPs. Importantly, the obtained characteristics demonstrate that the use of NADES as an alternative formulation component can yield chrysin-loaded chitosan nanosystems with physicochemical properties within the range reported for conventional TPP-based chitosan–chrysin nanoparticles [27,28]. This finding supports the potential of the NADES-assisted approach as an alternative strategy for the development of chitosan-based nanocarriers, while providing the additional formulation advantages associated with NADES-based systems.

#### 3.1.2. Encapsulation Efficiency (EE%)

The encapsulation efficiency (EE%) of CS-Chr NPs was determined by a UV–Vis spectrophotometric method based on the quantification of chrysin following complete disruption of the nanoparticle matrix in DMSO. The obtained EE% was 45.0 ± 1.5% (n = 3), indicating reproducible encapsulation of chrysin within the chitosan nanoparticle matrix. Based on the initial amount of chrysin used for nanoparticle preparation (2.72 mg), this corresponds to approximately 1.22 mg of encapsulated chrysin per 10 mL of nanoparticle dispersion. The successful encapsulation of chrysin in chitosan nanoparticles is consistent with previous findings reported by Siddhardha et al., who developed chrysin-loaded chitosan nanoparticles using ionic gelation with tripolyphosphate (TPP) as a crosslinking agent and reported an encapsulation efficiency of 80.86 ± 0.30% [27]. Although the EE% obtained in the present study was lower, the comparison is noteworthy because chrysin was successfully encapsulated in chitosan nanoparticles without the use of TPP. In the present formulation, NADES was incorporated during nanoparticle preparation, providing an alternative TPP-free approach for the development of chrysin-loaded chitosan nanoparticles. These findings demonstrate the feasibility of employing NADES as a formulation component for chrysin encapsulation in chitosan nanoparticles.

#### 3.1.3. TEM Analysis

**Figure 5:**
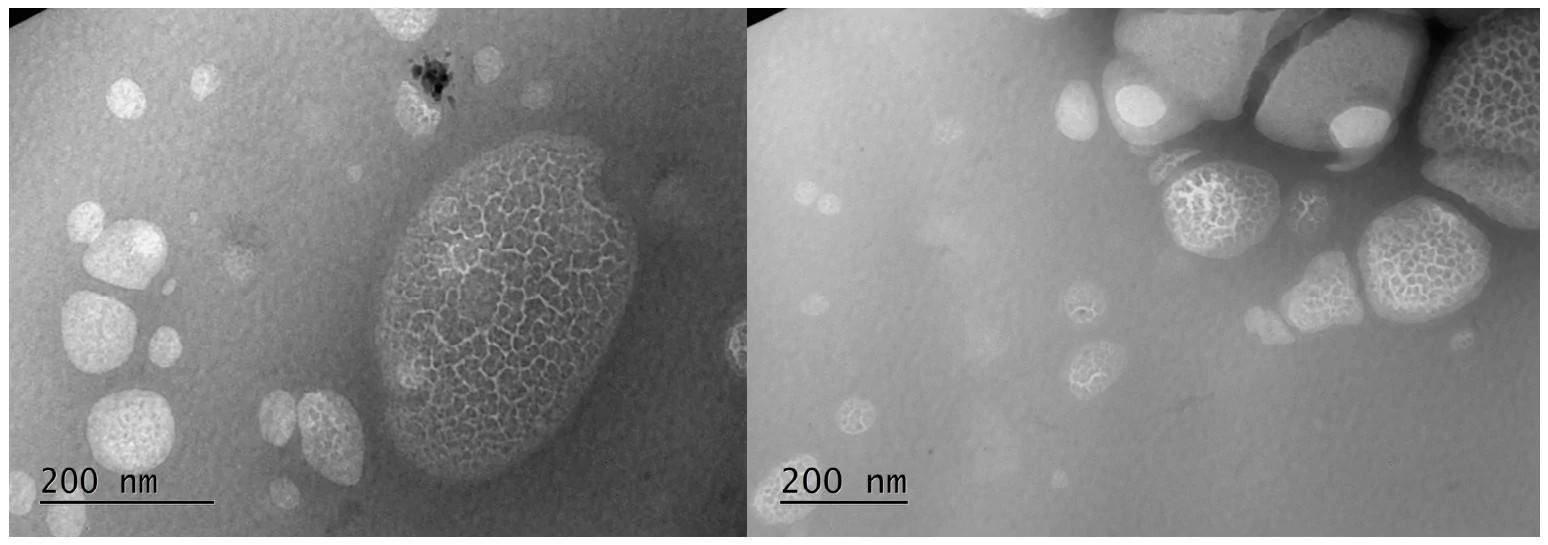
Image of the CS-Chr NPs through the Tranmission Electron Microscopy (TEM).

The morphology of the CS-Chr NPs was investigated by Transmission Electron Microscopy (TEM). The obtained TEM images revealed that the nanoparticles exhibited a predominantly spherical morphology with a smooth surface, as shown in Figure 5.

The particle size distribution observed by TEM was within the nanoscale range (approximately 50–200 nm). The particle sizes observed by TEM were broadly consistent with the hydrodynamic diameter determined by Nanoparticle Tracking Analysis (NTA), supporting the successful formation of nanosized CS-Chr NPs. However, the relatively high PDI values (0.565 for freshly prepared nanoparticles and 0.630 after 8 months of storage) indicate a heterogeneous particle size distribution. In addition, the increase in hydrodynamic diameter from 138.8 ± 5.3 nm to 194.7 ± 28.5 nm during storage suggests some degree of particle growth or association over time. Therefore, the developed nanoparticles should not be described as having a uniform particle size distribution or as being completely free from aggregation. Nevertheless, the nanoparticles remained within the nanoscale range during the investigated storage period, and the observed changes should be considered when evaluating their storage stability.

#### 3.1.4. In Vitro Release Study of Chrysin from CS-Chr NPs

**Figure 6:**
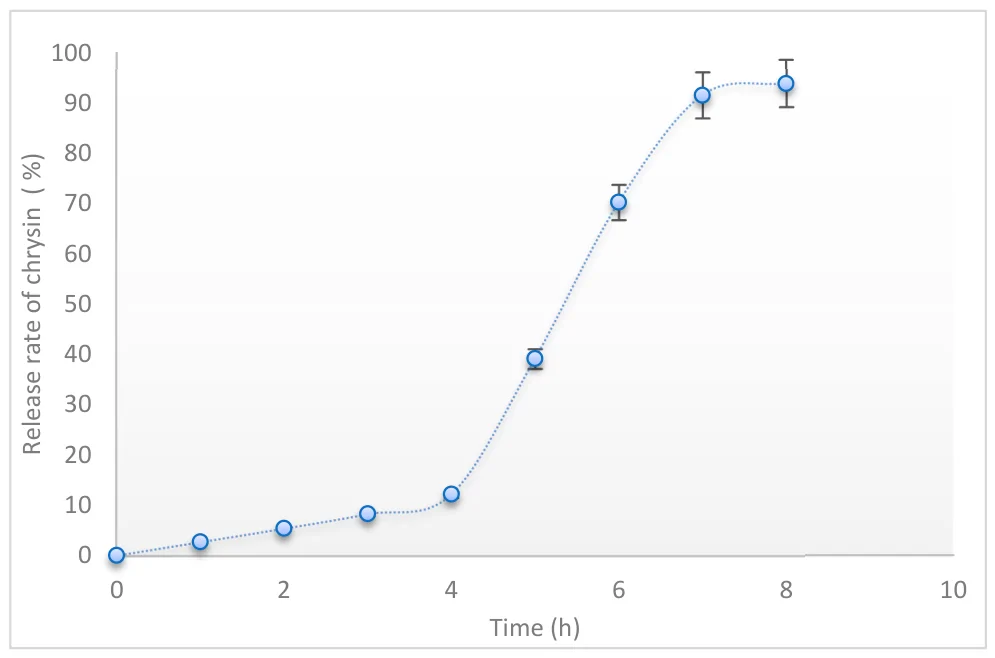
In vitro release profile of chrysin from CS-Chr NPs at 37 °C and pH 7.4.

The in vitro release profile of chrysin from the CS-Chr NPs at 37 °C and pH 7.4 is presented in Figure 6. The nanoparticles exhibited a biphasic release profile, characterized by a relatively low initial release followed by a pronounced increase in chrysin release. Approximately 5% of the encapsulated chrysin was released within the first 2 h, increasing to 39% after 5 h and reaching approximately 94% after 8 h. The limited release observed during the initial phase may be attributed to the strong association of chrysin with the chitosan matrix and its efficient entrapment within the nanoparticles. The subsequent increase in cumulative release is associated with the gradual diffusion of chrysin from the nanoparticle matrix into the surrounding release medium.

The obtained experimental release profile data were further analyzed by mathematical studies according to zero order, first order, Higuchi and Korsmeyer–Peppas models, which have been developed to describe the release kinetics and mechanism of a drug from an encapsulation matrix.

The correlation coefficients from each model (R^2^) were compared (Table 3). Among the kinetic models evaluated, the first-order model exhibited the highest correlation coefficient (R^2^ = 0.9828), indicating the best fit to the experimental release data. This result suggests that the release rate of chrysin was related to the amount of drug remaining within the nanoparticle system. Therefore, the first-order model was considered the most appropriate model for describing the overall release kinetics of chrysin from the CS-Chr NPs.

The Korsmeyer–Peppas model was additionally applied to gain further insight into the release mechanism. As this semi-empirical model is generally applied to the initial portion of the release profile, corresponding to up to 60% of the cumulative release, the release exponent (n) was determined from the slope of the corresponding linear relationship. The obtained n value was lower than 0.5 (n = 0.4099), indicating a Fickian diffusion-controlled release mechanism. Thus, the Korsmeyer–Peppas analysis indicates that diffusion was the predominant mechanism governing chrysin release from the CS-Chr NPs.

The release profile observed in the present study is comparable to previously reported findings for chrysin-loaded chitosan-based nanoparticles. Siddhardha et al. reported an initial burst release of approximately 36% within the first 2 h, followed by a more sustained release phase, with approximately 90.5% of chrysin released after 10 h [27]. In comparison, the CS-Chr NPs developed in the present study exhibited a substantially lower initial release, with only approximately 5% of chrysin released after 2 h, followed by a marked increase in release, reaching approximately 94% after 8 h. The lower initial release observed in the present formulation suggests more effective retention of chrysin within the chitosan nanoparticle matrix during the initial stage of the study. Differences in chrysin loading, nanoparticle composition, drug–polymer interactions, particle characteristics, and experimental release conditions may account for the differences observed between the present and previously reported formulations. In addition, the presence of NADES may influence the physicochemical characteristics of the chitosan nanoparticles and the solubilization of chrysin, potentially affecting the drug release behavior of the developed nanosystems.

#### 3.1.5. Hemolytic Assay

**Figure 7:**
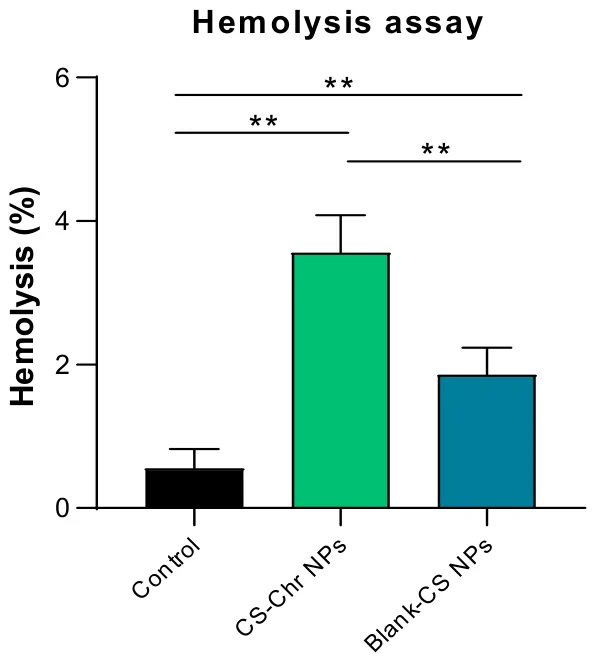
Hemolytic activity of chitosan-based nanoparticles. Hemolysis (%) was evaluated in u untreated control samples and in samples treated with CS-Chr NPs or Blank-CS NPs. Data are presented as mean ± SD from 10 paired samples (n = 10). Pairwise comparisons were performed using the Wilcoxon matched-pairs signed-rank test. CS-Chr NPs significantly increased hemolysis compared with both the untreated control (*p* = 0.0039) and Blank-CS NPs (*p* = 0.0039). Blank-CS NPs also induced significantly higher hemolysis than the untreated control (*p* = 0.0020). ** *p* < 0.001.

A hemolysis assay was performed to evaluate the interaction of the developed chitosan-based nanoparticles with the erythrocyte membrane [23,24]. As shown in Figure 7, both formulations induced relatively low levels of hemolysis under the experimental conditions. CS–Chr NPs, corresponding to a final chrysin concentration of 6 µg/mL, produced the highest hemolytic response, with a mean hemolysis value of approximately 3.5%. This value was significantly higher than that observed in both the control (*p* = 0.0039) and the Blank-CS NP group (*p* = 0.0039). Blank-CS NPs induced a lower mean hemolysis value of approximately 1.8%, which was nevertheless significantly higher than that of the control (*p* = 0.0020).

When interpreted according to commonly applied hemolysis criteria, values below 2% are considered non-hemolytic, values between 2% and 5% are considered slightly hemolytic, and values above 5% indicate hemolytic activity [30]. Accordingly, Blank-CS NPs were within the non-hemolytic range, whereas CS–Chr NPs were within the slightly hemolytic range. Nevertheless, both formulations remained below the 5% threshold generally used to identify significant nanoparticle-induced erythrocyte damage. Thus, although chrysin loading produced a statistically significant increase in hemolysis, the magnitude of the observed response remained relatively low under the tested concentration and exposure conditions.

### 3.2. Investigation of the Key Parameters Influencing Nanocomposite Eutectogel Formulation

The main influences of three selected independent variables (factors A, B and C) as the most important on the swelling performance of the nanocomposite eutectogel formulations was studied using a Box–Behnken experimental design. This response surface methodology was selected to evaluate the individual effects and interactions among the critical formulation parameters while minimizing the number of experimental trials. A total of 15 experimental runs were performed in triplicate, including three replicates of the central point to estimate the experimental error and improve the reliability of the developed model.

Specifically:

Factor A: The concentration of xanthan gum (% *w*/*v*) used for the preparation of the eutectogel. Xanthan gum is a natural hydrophilic polysaccharide extensively used in hydrogel-based delivery systems due to its excellent swelling ratio, biocompatibility, and ability to form three-dimensional polymeric networks [31]. The concentration of xanthan gum is a critical parameter affecting the network architecture, porosity, mechanical properties, and swelling behavior of hydrogel systems [32,33]. Previous studies have demonstrated that increasing xanthan gum concentration can modify the swelling capacity due to changes in polymer chain organization and water accessibility within the hydrogel matrix [34]. Therefore, xanthan gum concentration was selected as Factor A, with boundaries established between 10% (*w*/*v*) and 20% (*w*/*v*) [35].

Factor B: The chrysin content (% *w*/*w*, relative to the mass of chitosan) in the CS-Chr NPs incorporated into the eutectogel formulation was selected as an independent variable. This parameter was defined as the mass of chrysin relative to the initial mass of chitosan used for nanoparticle preparation. The chrysin content within the CS-Chr NPs was selected as an independent variable due to the bioactive properties of chrysin and its ability to interact with polymeric carriers through hydrogen bonding and other intermolecular interactions [27]. The amount of encapsulated chrysin may influence the physicochemical characteristics of the nanocomposite eutectogel by affecting the interactions between chrysin, the CS-Chr NPs system, and the xanthan gum polymeric network. Previous studies have demonstrated that chrysin incorporation into chitosan-based nanoparticles involves interactions between the hydroxyl groups of chrysin and the functional groups of chitosan, which can influence nanoparticle characteristics, encapsulation efficiency, and drug loading behavior [27]. Therefore, the chrysin concentration within the nanoparticles was investigated as Factor B, with the experimental boundaries established between 10% (*w*/*w*) and 30% (*w*/*w*).

Factor C: The concentration of CS-Chr NPs dispersion (% *v*/*v*). The concentration of nanoparticle dispersion incorporated into the eutectogel matrix represents an important formulation parameter, as nanoparticles can modify the internal structure of polymeric networks through polymer–nanoparticle interactions. Chitosan nanoparticles have been widely investigated as nanocarriers in drug delivery systems due to their biocompatibility, positive surface charge, and ability to control the release of encapsulated compounds [36]. Moreover, the incorporation of nanoparticles into hydrogel matrices can influence swelling behavior, porosity, and mechanical properties by altering the density and organization of the polymeric network [37]. Therefore, CS NPs dispersion concentration was selected as Factor C, with limits ranging from 10% (*v*/*v*) to 20% (*v*/*v*).

The swelling ratio (%) at 3 h was selected as the response parameter (R1), as it represents an essential characteristic for evaluating the hydration capacity, structural integrity, and potential drug delivery performance of eutectogel systems [32]. A total of 15 experiments were performed according to the Box–Behnken experimental design, as shown in Table 4. Three center-point replicates were selected to estimate the pure experimental error. Following the collection of response data, statistical analysis was performed, and a mathematical model was developed to describe the relationship between the selected formulation variables and the swelling behavior of the nanocomposite eutectogel.

### Swelling Ratio

The experimental response was modeled as a polynomial equation to describe the effects of the independent variables (A, B, and C) on the response variable (R1). In this model, the independent variables were defined as follows: Factor A corresponds to the xanthan gum concentration (% *w*/*v*) used for the preparation of the eutectogel, Factor B represents the chrysin (Chr) loading concentration (% *w*/*w*) within the CS-Chr NPs incorporated into the eutectogel formulation, and Factor C corresponds to the concentration of CS-Chr NPs dispersion (% *v*/*v*).

While the data were initially fitted to a second-order polynomial equation, the analysis indicated the significance of higher-order interactions. Thus. the selected modified model incorporates linear terms (A, B, C), two-factor interaction terms (AB, BC), quadratic terms (A^2^, B^2^, C^2^) together with a specific third-order interaction term (AB^2^) as described by the following coded and actual equations (Equations (4) and (5), respectively):

(4) Swelling Ratio (R1) = 36.67 + 35.50A + 6.75B − 8.25C − 19.00AB + 7.00BC + 9.42A^2^ + 20.92B^2^ + 32.42C^2^ − 18.50AB^2^

(5) Swelling Ratio (R1) = 551.58333 − 11.40000 Xanthan Gum − 26.29167 Chr − 43.35000 CS NPs + 1.10000 Xanthan Gum Chr + 0.140000 Chr CS NPs + 0.376667 (Xanthan Gum)^2^ + 0.764167 Chr^2^ + 1.29667 (CS NPs)^2^ − 0.037000 Xanthan Gum Chr^2^

More specifically, the initial response surface model was formulated as a second-order polynomial, including the linear terms (A, B, C), quadratic terms (A^2^, B^2^, C^2^) and two-factor interaction terms (AB, AC and BC). Although the initially selected quadratic model was significant (*p*-value = 0.0185) and showed a satisfactory R^2^ (R^2^ = 0.9327) and an adjusted R^2^ of 0.8115, the model exhibited a statistically significant lack of fit (*p*-value = 0.0258 < 0.05). Therefore, based on the *p*-values of the individual model terms and the lack-of-fit assessment, modifications to the initial model were considered necessary, and the model terms were re-evaluated through analysis of variance. Following this assessment, the interaction term AC was excluded from the final model as non-significant, since showed a *p*-value of 0.5899 (which is greater than 0.1000). After this modification, the model still exhibited a statistically significant lack of fit. Then, the model was further extended by incorporating the statistically significant third-order interaction term AB^2^ (*p*-value = 0.0255), which was found to provide a significant contribution to the model (model *p*-value = 0.0016) with a non-significant lack of fit (*p*-value = 0.0714). Furthermore, the modified model showed an improvement in both the R^2^ (0.9759) and the Adjusted R^2^ (0.9326) values compared to the initially studied quadratic model.

Regarding the lack of fit of the modified model, was not statistically significant (*p*-value = 0.0714 > 0.05), however the relatively low *p*-value (<0.1) suggests that the predictive performance should be interpreted with some caution.

This observed lack of fit value may be associated with the inherent variability of the swelling response of the eutectogel, which can arise from small differences in the internal structure, crosslinking density, and hydration behavior of the hydrogel matrix between experimental runs. Such variability may be particularly pronounced for swelling measurements, as minor differences in matrix organization and water uptake can result in appreciable differences in the measured swelling ratio. Significant lack of fit has been reported in response surface optimization studies of hydrogel systems, and was attributed to experimental variability and heterogeneity of the investigated hydrogel samples under certain formulation conditions [38].

Therefore, although the modified model adequately captured the main relationships between the formulation factors and the swelling response within the selected investigated experimental boundaries, some experimental variation may remain unexplained by the fitted model.

The Adeq Precision value of 14.4354, being greater than the desirable value of 4, indicated an adequate signal-to-noise ratio and supported the use of the model for exploring the design space. According to the ANOVA results (Table 5), xanthan gum concentration (factor A) was the most influential factor affecting the swelling ratio, exhibiting a highly significant *p*-value (*p*-value = 0.0004) and the highest F-value of 72.93. The significant quadratic effects were also observed for CS NPs dispersion (C^2^, *p* = 0.0007, F-value = 56.12) and chrysin loading (B^2^, *p* = 0.0047, F-value = 23.37) together with the effect of the interaction between xanthan gum and chrysin loading (AB, *p* = 0.0060, F-value = 20.89) further indicate that the swelling response is governed by nonlinear and formulation-dependent effects. These findings highlight the importance of considering the combined effects of formulation variables when interpreting the swelling response.

The 3D response surface plots illustrate the relationships between the investigated formulation factors and the selected response variable (swelling ratio, %), providing insight into the individual and interactive effects of the independent variables on the performance of the nanocomposite eutectogel system.

**Figure 8:**
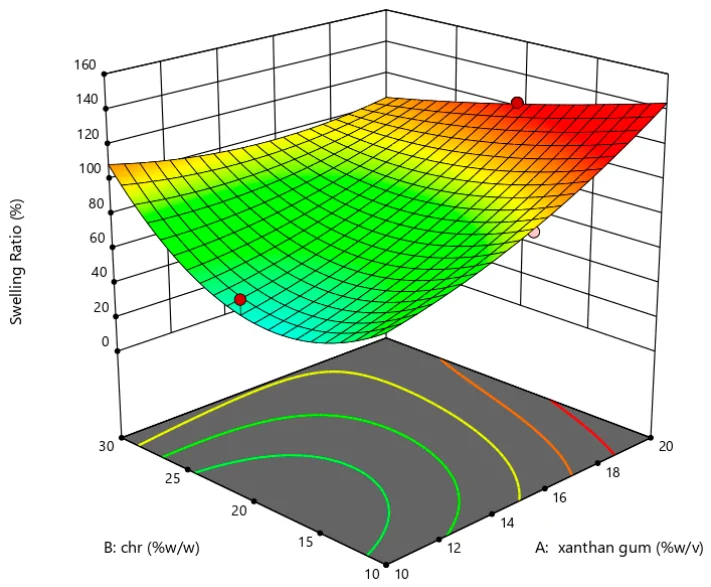
RSM plots for swelling ratio (%) response (R1) showing the correlation between the swelling ratio and the factors: xanthan gum (% *w*/*v*) and Chr (% *w*/*w*) (CS NPs = 10% *v*/*v*). The color scale represents the predicted response values, with blue indicating lower values and red indicating higher values.

According to the response surface methodology (RSM) plot (Figure 8), while keeping the CS NPs dispersion constant at 10% (*v*/*v*), the highest swelling ratios were achieved using higher xanthan gum concentrations (>18% *w*/*v*) in combination with lower chrysin loading (<15% *w*/*w*). Increasing the xanthan gum concentration enhanced the swelling capacity of the eutectogel, whereas increasing chrysin loading resulted in a gradual decrease in the swelling ratio.

**Figure 9:**
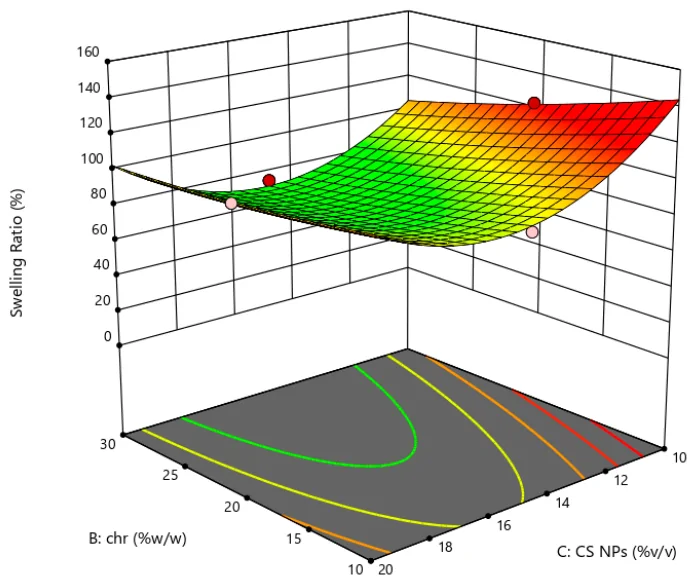
RSM plots for swelling ratio (%) response (R1) showing the correlation between the swelling ratio and the factors: Chr (% *w*/*w*) and CS NPs (% *v*/*v*) (xanthan gum = 20% *w*/*v*). The color scale represents the predicted response values, with blue indicating lower values and red indicating higher values.

Based on the RSM plot (Figure 9), while keeping the xanthan gum concentration constant at 20% (*w*/*v*), the highest swelling ratios were achieved at lower CS NPs dispersion levels (<12% *v*/*v*) combined with lower chrysin loading (<15% *w*/*w*). Increasing the CS NPs concentration gradually decreased the swelling ratio, suggesting that excessive nanoparticle incorporation limited the water uptake capacity of the eutectogel. Similarly, increasing the chrysin loading beyond values around 15% *w*/*w* resulted in a reduction in the swelling response.

Therefore, lower nanoparticle dispersion levels combined with low-to-moderate chrysin loading were identified as most favorable conditions for achieving the maximum swelling ratio.

To experimentally assess the selected formulation conditions, three additional confirmation experiments were performed under the selected conditions (xanthan gum: 20% *v*/*v*, chrysin loading: 13.6% and CS NPs: 10% *v*/*v*), and the results of the swelling ratio are presented in Table 6. According to the confirmation experiments results, the swelling ratio fells within the corresponding 95% prediction intervals calculated by the fitted model. These results provided experimental support for the selected formulation conditions; however, considering the lack of fit value (*p*-values = 0.07), the predictive capability of the model should be interpreted with caution.

### 3.3. Fourier-Transform Infrared Spectroscopy (FT-IR)

**Figure 10:**
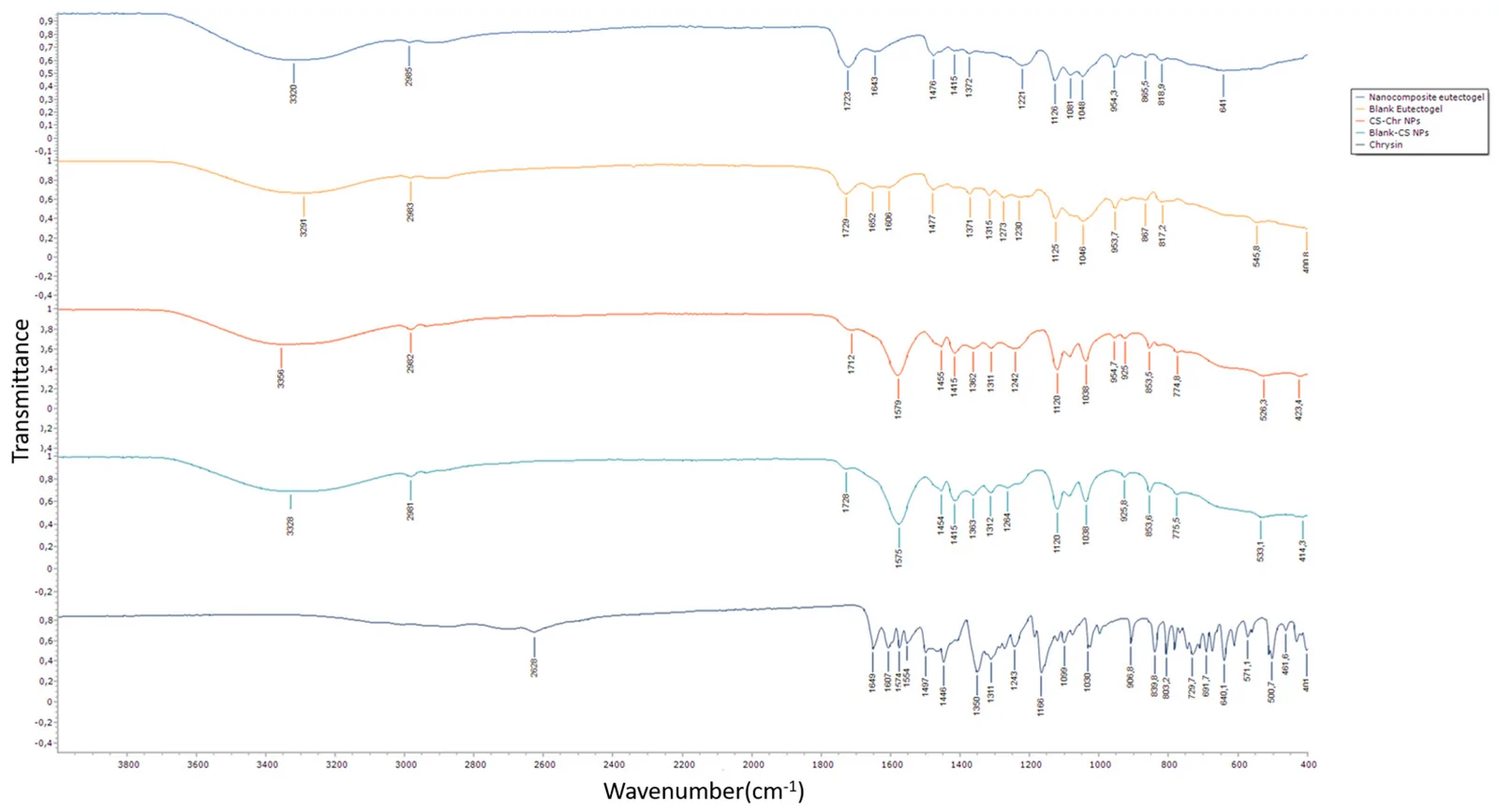
FT-IR spectraof nanocomposite eutectogel, blank eutectogel, CS-Chr NPs, Blank-CS NPs, chrysin.

Fourier-transform infrared spectroscopy (FT-IR) analysis was performed to investigate the intermolecular interactions of the developed CS-Chr NPs, Blank-CS-NPs, nanocomposite eutectogel system and blank eutectogel (Figure 10 and Table 7).

The FT-IR spectra of chrysin and the investigated formulations are presented in Figure 10, while the characteristic absorption bands and their assignments are summarized in Table 7. The FT-IR spectrum of chrysin exhibited the characteristic absorption bands associated with its flavonoid structure. In particular, the band at 1649 cm^−1^ can be attributed to the conjugated carbonyl (C=O) stretching vibration, while the bands at 1574 and 1446 cm^−1^ are associated with vibrations of the aromatic ring system. Additional absorptions observed in the fingerprint region, including those at 1350, 1243, 1166, 1099, and 1030 cm^−1^, are related mainly to C–O, C–O–C, and aromatic skeletal vibrations. These characteristic absorptions provide the reference spectral profile for evaluating the incorporation of chrysin into the chitosan-based nanoparticles and its subsequent integration into the eutectogel matrix [27].

The FT-IR spectrum of the Blank-CS NPs exhibited the characteristic absorption profile of the chitosan-based system. The broad band centered at 3324 cm^−1^ was assigned to the overlapping O–H and N–H stretching vibrations, reflecting the hydrophilic nature of chitosan and the extensive hydrogen-bonding interactions within the matrix. The band at 2981 cm^−1^ was attributed to C–H stretching vibrations. The absorption at 1575 cm^−1^ can be associated with N–H bending vibrations of the protonated amino groups of chitosan, while the bands observed at 1362, 1264, 1120, and 1038 cm^−1^ were assigned mainly to C–O, C–O–C, and C–O stretching vibrations of the polysaccharide backbone. The additional absorption at 1728 cm^−1^ may be related to carbonyl-containing components associated with the formulation, particularly residual lactic acid/NADES-derived contributions. Overall, the observed spectral profile is consistent with the characteristic FT-IR features of chitosan-based systems [39].

The spectrum of the CS-Chr NPs retained the main absorption bands of the Blank-CS NPs, while several changes in band position and relative intensity were observed. The broad O–H/N–H stretching band shifted from 3324 cm^−1^ in the Blank-CS NPs to 3356 cm^−1^ in the CS-Chr NPs, while the carbonyl-related absorption shifted from 1728 to 1712 cm^−1^. In addition, changes were observed in the fingerprint region, including the bands at 1456, 1362, and 1242 cm^−1^. These shifts and changes in band profiles indicate modifications of the local molecular environment following chrysin incorporation and are consistent with intermolecular interactions between chrysin and the functional groups of chitosan. In particular, hydrogen-bonding interactions between the phenolic hydroxyl groups of chrysin and the hydroxyl and amino groups of chitosan may contribute to the observed changes in the O–H/N–H stretching region. Similar spectral changes have been reported for chrysin-loaded chitosan nanoparticles and have been associated with interactions between chrysin and the chitosan matrix [27]. Importantly, no distinct new absorption band corresponding to the formation of a new covalent functional group was detected. Therefore, the FT-IR results support the incorporation of chrysin within the chitosan nanoparticle matrix predominantly through intermolecular interactions, without evidence of major chemical transformation of the chrysin structure.

The blank eutectogel exhibited the characteristic spectral features of the NADES/xanthan-gum-based matrix. The broad absorption centered at 3291 cm^−1^ was attributed to O–H stretching vibrations and reflects the extensive hydrogen-bonding network established between the hydroxyl-containing components of the NADES and the xanthan gum polysaccharide. The band at 2983 cm^−1^ was associated with C–H stretching, whereas the absorption at 1729 cm^−1^ was attributed to C=O stretching associated mainly with the lactic acid-containing eutectic system. The bands at 1606 and 1477 cm^−1^, together with those at 1371, 1230, 1125, and 1046 cm^−1^, were assigned to the various skeletal, C–O–C, and C–O vibrations of the formulation components. The observed spectral profile is consistent with the coexistence of the NADES components and the polysaccharide network and supports the formation of an extensively hydrogen-bonded eutectogel structure [35].

In the nanocomposite eutectogel, the FT-IR profile retained the main spectral features of the blank eutectogel, while distinct changes in band positions were observed. The broad O–H/N–H stretching region appeared at 3320 cm^−1^, compared with 3291 cm^−1^ for the blank eutectogel, indicating changes in the hydrogen-bonding environment following incorporation of the CS-Chr NPs. The carbonyl-related band also shifted from 1729 to 1723 cm^−1^. Furthermore, the band observed at 1606 cm^−1^ in the blank eutectogel shifted to 1643 cm^−1^ in the nanocomposite system, accompanied by changes in the fingerprint region, particularly at 1221 and 1048 cm^−1^. These spectral modifications indicate changes in the molecular environment of the functional groups following incorporation of the chrysin-loaded chitosan nanoparticles and are consistent with additional intermolecular interactions among the xanthan gum matrix, NADES components, chitosan, and chrysin.

Importantly, the characteristic chrysin-related bands were not observed as completely unchanged peaks in the nanocomposite eutectogel; rather, their spectral contributions appear to overlap with and/or undergo shifts within the complex polymer/NADES matrix. In particular, the shift in the band from 1606 to 1643 cm^−1^, together with the changes observed in the O–H/N–H stretching region and at 1221 and 1048 cm^−1^, indicates modifications in the molecular environment of the corresponding functional groups following incorporation of the CS-Chr NPs. These spectral changes are consistent with intermolecular interactions among the xanthan gum matrix, NADES components, chitosan, and chrysin. The absence of a clearly new absorption band attributable to a newly formed covalent functional group suggests that the incorporation of the CS-Chr NPs does not result in the formation of a new dominant covalent chemical structure.

### 3.4. Thermogravimetric Analysis (TGA)

**Figure 11:**
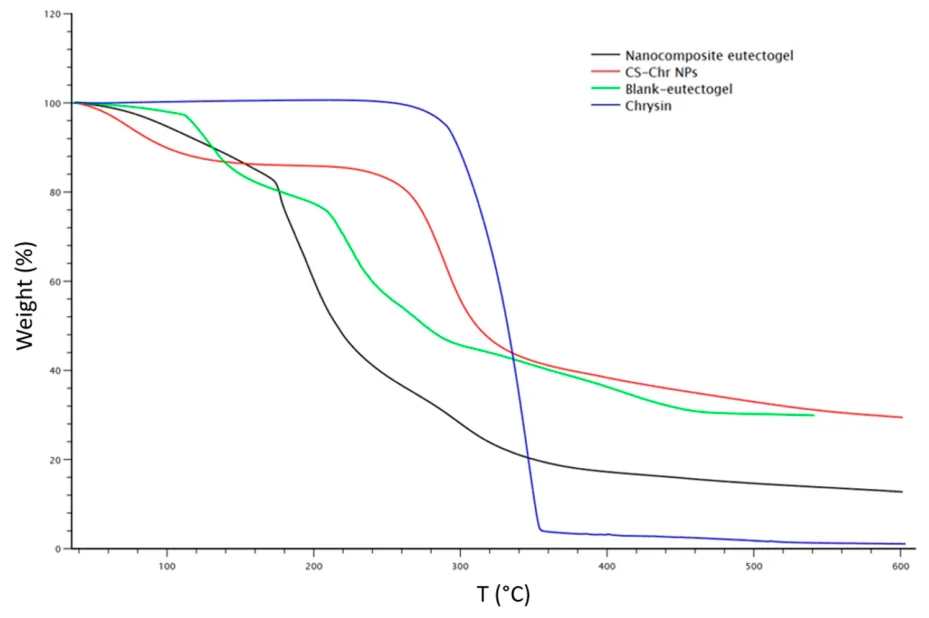
Combined diagram of nanocomposite eutectogel (black), CS-Chr NPs (red), Blank-eutectogel (green), Chrysin (blue).

Thermogravimetric analysis (TGA) was performed to investigate the thermal behavior and stability of pure chrysin, CS-Chr NPs, blank eutectogel, and the developed nanocomposite eutectogel system (Figure 11).

Pure chrysin exhibited a characteristic single-step thermal degradation profile, with the maximum degradation rate occurring at 342 °C, corresponding to the decomposition of its flavonoid structure. This degradation event is mainly associated with the cleavage of the aromatic backbone and the breakdown of oxygen-containing functional groups. The absence of additional degradation stages indicates a homogeneous thermal decomposition pathway for the free bioactive compound. The TGA curve showed an almost complete 98.9% mass loss, leaving only approximately 1.1% residual mass at 600 °C, confirming the nearly complete thermal decomposition of chrysin under the applied conditions and the absence of thermally stable degradation products [40].

The CS-Chr NPs exhibited a multistep degradation profile, reflecting the combined thermal behavior of the chitosan nanoparticle matrix and the organic components incorporated into the system. An initial weight loss of approximately 4.1% below 70 °C was attributed to the evaporation of physically adsorbed water, followed by a second degradation stage (69–193 °C, 9.3% mass loss) associated with the removal of bound water and the NADES constituents (LA and ChCl). In our previous study, we have shown that the decomposition of the pure NADES ChCl:LA happens at 194 °C; therefore, in the case of CS-Chr NPs studied here, this degradation temperature can be regarded as an indication of the presence of the NADES in the final nanoformulation [41]. The major degradation step occurred between 200 and 580 °C, accounting for approximately 55.9% mass loss, and corresponded to the thermal decomposition of the chitosan polysaccharide backbone through dehydration, depolymerization, and glycosidic bond cleavage. The maximum degradation rate was observed at approximately 290 °C, representing the principal decomposition stage of the chitosan/NADES hybrid network. Compared with free chrysin, which exhibited a distinct degradation peak at 342 °C, the absence of an independent chrysin degradation peak confirms its successful encapsulation in chitosan [42,43].

The blank eutectogel exhibited a multistage degradation profile resulting from the combined thermal response of the NADES phase and the xanthan gum polymeric network. Six degradation regions were identified at 39–137 °C, 138–204 °C, 205–282 °C, 284–338 °C, 339–402 °C, and 403–594 °C. The initial weight loss (4.8%) was mainly attributed to the evaporation of free and bound water molecules. The subsequent degradation stages corresponded to the decomposition of the NADES components and the progressive thermal degradation of the xanthan gum polymeric network through cleavage of glycosidic linkages and carbonization reactions. The maximum degradation rate of the eutectogel matrix was observed at approximately 200 °C, while a relatively high residual mass of approximately 30% at 600 °C indicated the formation of thermally stable carbonaceous products, reflecting the contribution of the NADES phase to the thermal stability of the polymeric matrix. These findings are consistent with previous reports describing the multistep thermal degradation of NADES-based polysaccharide systems [44,45].

The nanocomposite eutectogel exhibited a broad multistep degradation profile, confirming the formation of an integrated hybrid network composed of xanthan gum, CS-Chr NPs, and the NADES phase. The absence of a distinct chrysin degradation peak indicates its successful incorporation and stabilization within the matrix through intermolecular interactions. The main degradation stage occurred between 99 and 262 °C (59.0% mass loss), followed by further decomposition from 263 to 590 °C (22.8% mass loss). The lower residual mass of the nanocomposite eutectogel (~13% at 600 °C) compared with the blank eutectogel (~30%) suggests reduced char formation due to the presence of CS-Chr NPs and chrysin, which modified the degradation pathway and limited extensive carbonization [32,46].

Overall, the TGA results confirmed the successful incorporation of CS-Chr NPs into the eutectogel network. The modified degradation profile, together with the absence of an independent chrysin degradation peak, demonstrated the effective encapsulation and stabilization of the bioactive compound within the hybrid matrix. These findings highlight the role of the interconnected xanthan/chitosan/NADES network in modulating the thermal degradation pathway and support the potential of the eutectogel system as a stable delivery platform.

### 3.5. In Vitro Release Study of Chrysin from the Nanocomposite Eutectogel

**Figure 12:**
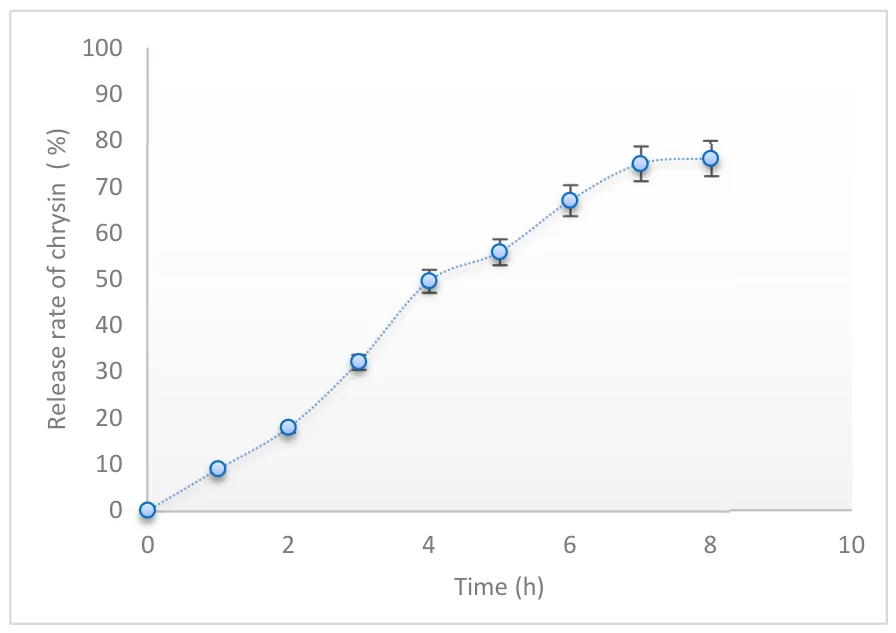
In vitro release profile of chrysin from nanocomposite eutectogel at 37 °C and pH 7.4.

The in vitro release profile of chrysin from the nanocomposite eutectogel at 37 °C and pH 7.4 is presented in Figure 12. The nanocomposite eutectogel exhibited a sustained release profile, with approximately 18% of the encapsulated chrysin released within the first 2 h, increasing to 56% after 5 h and reaching approximately 76% after 8 h. The gradual release may be attributed to the controlled diffusion of chrysin through the three-dimensional eutectogel network, which acts as an additional diffusion barrier and restricts the mobility of the incorporated CS-Chr NPs. Swelling and relaxation of the polymeric network may also contribute to the progressive release.

Compared with the CS-Chr NPs described in Section 3.1.4, which released approximately 5%, 39%, and 94% of chrysin after 2, 5, and 8 h, respectively, the nanocomposite eutectogel exhibited a more sustained release behavior. Although the initial release was higher at 2 h (18% versus 5%), the subsequent release rate was slower, resulting in substantially lower cumulative release at 8 h (76% versus 94%). This behavior indicates that incorporation of the CS-Chr NPs into the eutectogel matrix effectively prolonged chrysin release.

The release kinetics were evaluated using zero-order, first-order, Higuchi, and Korsmeyer–Peppas models (Table 8). The Higuchi model showed the best fit (R^2^ = 0.9796), indicating that diffusion was the predominant mechanism governing chrysin release. This finding is consistent with the study of Anuța et al. (2025) [47], who reported sustained drug release from xanthan gum–hyaluronic acid eutectogels, with high Higuchi correlation coefficients (R^2^ > 0.99). Moreover, the Korsmeyer–Peppas n values (0.507–0.638) indicated anomalous transport, involving both diffusion and polymer relaxation and/or swelling [47]. Similarly, the n value of 0.7247 determined in the present study, indicates a non-Fickian (anomalous) release mechanism. Thus, although diffusion was the predominant mechanism, relaxation and/or swelling of the eutectogel network also contributed to chrysin release.

Overall, incorporation of CS-Chr NPs into the xanthan-gum-based eutectogel resulted in a more controlled and sustained release compared with the one observed from the CS-Chr NPs, demonstrating the potential of the developed system for prolonged chrysin delivery.

## 4. Conclusions

This study presents the successful development of a sustainable hybrid nanocomposite eutectogel platform for the encapsulation and delivery of chrysin, combining chrysin-loaded chitosan nanoparticles (CS-Chr NPs) with a xanthan-gum-based eutectogel structured by a choline chloride/lactic acid (ChCl/LA) natural deep eutectic solvent (NADES). A key innovation of the proposed system is the multifunctional role of the ChCl/LA NADES, which served simultaneously as (i) the component that promoted the formation and stabilization of CS-Chr NPs through molecular interactions with the chitosan matrix, eliminating the need for conventional external crosslinkers such as sodium tripolyphosphate (TPP), and (ii) the eutectic liquid phase immobilized within the xanthan gum polymeric network, where xanthan gum acted as the network-forming/gelating agent to generate the eutectogel.

The developed CS-Chr NPs exhibited a nanoscale hydrodynamic diameter of 138.8 nm, as determined by nanoparticle tracking analysis, together with a high positive ζ-potential of +46.53 mV and a predominantly spherical morphology. Notably, the nanoparticles maintained a positive ζ-potential after 8 months of storage at 4 °C (+42.83 ± 2.13 mV); however, an increase in hydrodynamic diameter from 138.8 ± 5.3 to 194.7 ± 28.5 nm was observed, accompanied by an increase in PDI from 0.565 ± 0.06 to 0.630 ± 0.04, indicating increased particle-size heterogeneity during storage. Furthermore, an encapsulation efficiency of approximately 45% was achieved, demonstrating the ability of the developed nanocarrier system to effectively encapsulate the poorly water-soluble flavonoid chrysin. Hemocompatibility assessment provided preliminary evidence regarding the safety profile of the developed nanocarriers, as both blank and chrysin-loaded chitosan nanoparticles exhibited hemolysis levels below the 5% threshold under the tested conditions. Although chrysin incorporation resulted in a slight increase in hemolytic activity compared with the blank formulation, the observed values remained below this threshold, supporting the potential suitability of the developed nanoparticle system for biomedical applications.

The Box–Behnken experimental design provided a systematic approach for investigating the formulation factors governing the swelling behavior of the nanocomposite eutectogel. Xanthan gum concentration was identified as the most influential factor, while the significant quadratic effects of CS NPs dispersion and chrysin loading, together with the interaction between xanthan gum concentration and chrysin loading, highlighted the nonlinear and formulation-dependent nature of the swelling response. The selected formulation, comprising 20% (*w*/*v*) xanthan gum, 10% (*v*/*v*) CS-Chr NPs dispersion, and 13.6% (*w*/*w*) chrysin loading within the nanoparticles, exhibited a swelling ratio exceeding 120% under simulated gastric conditions (pH 1.2, 37 °C) while maintaining its structural integrity for at least 3 h. FTIR and TGA analyses further supported the successful incorporation of the chrysin-loaded nanoparticles into the eutectogel matrix and indicated the presence of intermolecular hydrogen-bonding interactions that may contribute to the enhanced structural stability of the hybrid system.

The release of chrysin from CS-Chr NPs reached 94% after 8 h, and exhibited predominantly Fickian diffusion (n = 0.4099). Incorporation of the CS-Chr NPs into the xanthan-gum-based eutectogel resulted in a more sustained chrysin release (76% after 8 h). The eutectogel release was predominantly diffusion-driven (Higuchi, R^2^ = 0.9796), with anomalous transport (n = 0.7247) indicating an additional contribution of polymer relaxation and/or swelling.

Overall, the developed NADES-based nanocomposite eutectogel represents a promising green delivery platform for poorly water-soluble bioactive compounds. By combining renewable biopolymers, biocompatible natural deep eutectic solvents, and a simplified nanoparticle fabrication process free of conventional crosslinkers, this work contributes to the advancement of sustainable nanotechnology and provides a versatile strategy for the development of environmentally friendly drug delivery systems with potential biomedical applications.

## Figures and Tables

**Table 1:** Design parameters for Box–Behnken design.

Independent Variables		Level		
Factors		Low (−1)	Medium (0)	High (1)
A	Xanthan gum (% *w*/*v*)	10	15	20
B	Chrysin (% *w*/*w*)	10	20	30
C	CS NPs (% *v*/*v*)	10	15	20
R1		Swelling Ratio (%)		

**Table 2:** Hydrodynamic diameter, concentration, Polydispersity Index (PDI) and ζ-potential of CS NPs.

Sample	Hydrodynamic Diameter (nm)	Polydispersity Index (PdI)	Concentration (Particles/mL)	ζ-Potential (mV)
Blank-CS NPs	134.6 ±7.3	0.520 ± 0.03	1.38 × 10^7^ ± 2.19 × 10^6^	40.61 ± 1.25
CS-Chr NPs	138.8 ± 5.3	0.565 ± 0.06	5.54 × 10^7^ ± 2.79 × 10^6^	46.53 ± 2.61
CS-Chr NPs (8-month stability, 4 °C)	194.7 ± 28.5	0.630 ± 0.04	5.71 × 10^7^ ± 6.73 × 10^6^	42.83 ± 2.13

**Table 3:** Results from kinetics of chrysin release from the CS-Chr NPs.

Zero Order	First Order	Higuchi	Korsmeyer–Peppas	First Order Equation	Korsmeyer–Peppas Equation
R^2^	R^2^	R^2^	R^2^	y = 0.2807x + 0.088	y = 0.4099 x − 0.064
0.8207	0.9828	0.8790	0.8693		

**Table 4:** Values of independent (A, B, C) and dependent (R1) variables of nanocomposite eutectogel.

Νο.	Factor A (% *w*/*v*)	Factor B (% *w*/*w*)	Factor C (% *v*/*v*)	R1 (%)
1	15	10	20	76
2	10	30	15	79
3	15	20	15	35
4	15	30	10	90
5	20	10	15	93
6	15	20	15	35
7	20	20	20	105
8	15	30	20	97
9	10	20	20	26
10	20	30	15	75
11	10	20	10	60
12	15	20	15	40
13	15	10	10	97
14	20	20	10	123
15	10	10	15	21

**Table 5:** ANOVA results.

Model	Lack-of-Fit	A	B	C	AB	BC	A^2^	B^2^	C^2^	AB^2^
*p*-value = 0.0016	0.0714	0.0004	0.0701	0.0377	0.0060	0.1530	0.0815	0.0047	0.0007	0.0255
F-value = 7.70	22.51	72.93	5.27	7.88	20.89	2.84	4.74	23.37	56.12	9.90
R^2^ = 0.9759, Adjusted R^2^ = 0.9326										

**Table 6:** Model validation at the 95% confidence level.

Response	Predicted Mean	Predicted Median	Std. Dev.	n	SE Pred	95% PI Low	Data Mean	95% PI High
Swelling Ratio	135.56	135.56	8.31465	3	8.46486	113.8	120	157.319

**Table 7:** Characteristic FT-IR absorption bands of chrysin, Blank-CS NPs, CS-Chr NPs, blank eutectogel, and nanocomposite eutectogel.

Band Origin	Chrysin	Blank-CS-NPs	CS-Chr-NPs	Blank Eutectogel	Nanocomposite Eutectogel
O–H/N–H stretching	—	3324	3356	3291	3320
C–H stretching	—	2981	2982	2983	2985
C=O stretching	—	1728	1712	1729	1723
C=O stretching of conjugated carbonyl group	1649	—	—	—	—
Aromatic C=C/N–H bending	1574	1575	1579	1606	1643
Aromatic skeletal vibrations/C–H bending	1446	1444	1456	1477	1476
C–O/C–H-related vibrations	1350	1362	1362	1371	1372
C–O/C–O–C stretching	1243	1264	1242	1230	1221
C–O–C/C–O stretching	1166	—	—	—	—
C–O–C stretching	1099	1120	1120	1125	1126
C–O stretching	1030	1038	1038	1046	1048
Aromatic C–H out-of-plane bending	898.8	925.8	862	867	865.5
Aromatic C–H out-of-plane bending	803.2	835.6	774.8	817.2	817.9

**Table 8:** Results from kinetics of chrysin release from the nanocomposite eutectogel.

Zero Order	First Order	Higuchi	Korsmeyer–Peppas	Higuchi Equation	Korsmeyer–Peppas Equation
R^2^	R^2^	R^2^	R^2^	y = 43.077x − 40.769	y = 0.7247x − 0.1006
0.9498	0.7483	0.9796	0.9637		

## Data Availability

The original contributions presented in this study are included in the article. Further inquiries can be directed to the corresponding author.
